# Finasteride induced Gynecomastia: Case report and Review of the Literature

**DOI:** 10.4103/0974-7753.51930

**Published:** 2009

**Authors:** Yuval Ramot, Tali Czarnowicki, Abraham Zlotogorski

**Affiliations:** Department of Dermatology, Hadassah-Hebrew University Medical Center, Jerusalem ‐ 912 00, Israel

**Keywords:** Androgenetic alopecia, finasteride, gynecomastia

## Abstract

Finasteride (1 mg/day) is widely utilized by dermatologists for the treatment of androgenetic alopecia. Although enjoying a relatively good safety profile, several sex-related adverse effects have been reported with this drug. Here we report two cases of gynecomastia, one of them bilateral, caused by Propecia^®^ prescribed for the treatment of androgenetic alopecia. Although relatively rare, physicians should be aware of this side effect and inform their patients when prescribing this medication.

## INTRODUCTION

Finasteride, 1 mg/day (Propecia^®^; MSD), a type-II 5α-reductase (5α-R) inhibitor, is the only approved treatment prescribed at present for androgenetic alopecia.[1] Finasteride 5 mg is another alternative, but usually sold off-label, and recently dutasteride, a dual 5α-R inhibitor, was reported as a potential and more effective alternative.[2] Propecia^®^ is marketed internationally as a drug with almost no side effects, and physicians routinely avoid discussion of its uncommon potential side effects. Here, we report two cases that developed gynecomastia while treated with Propecia^®^ and want to shed light on this side effect that seems more common and meaningful than previously reported.

## CASE REPORT

**Figure 1:**
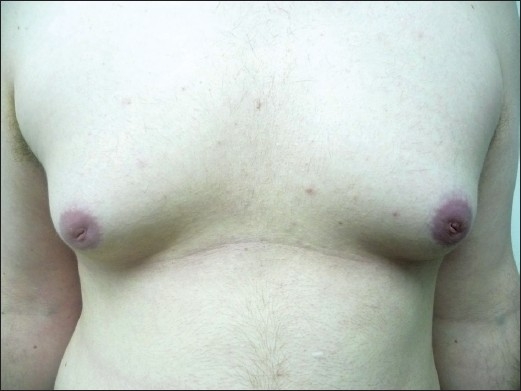
Bilateral gynecomastia, patient 1

A 21-year-old male, with androgenetic alopecia, generally healthy except for hypothyroidism treated with µg/d Eltroxin, developed bilateral gynecomastia four months after finasteride 1 mg/day initiation. Ten months after cessation of treatment, the patient still had enlarged breasts with no apparent improvement [Figure 1]. The second patient is a 65-year-old healthy male with androgenetic alopecia who developed unilateral left gynecomastia after two months of treatment. Stopping the treatment led to major improvement within two months, but at follow-up six years after this treatment, there is still slight residual swelling.

## DISCUSSION

Finasteride is a 4-aza-steroid that specifically inhibits the type II isoform of 5α-R, thereby decreasing the conversion of testosterone to its active metabolite dihydrotestosterone by 75–80%.[3] This inhibition leads to increased conversion of testosterone to estradiol and androstenediol in peripheral tissues (e.g., liver, testes, and peripheral blood). The increased estrogen levels may lead to sexually adverse events as was indeed shown in the largest trial reported on the use of finasteride 1 mg/day for men with androgenetic alopecia, where the only disturbances observed were decreased libido, difficulty in achieving erection, and decrease in semen′s amount.[4] Most of these side effects were claimed to be no more common than in control group.

Gynecomastia is a recognized side effect of a variety of conditions that lead to hormonal imbalance.[5] Among these drugs play a major cause, and exogenous estrogens, digoxin, phenothiazide, or propranolol have been commonly associated with this condition.[6] Finasteride use in the dose of 5 mg/day [usually used for the treatment of benign prostatic hyperplasia, (BPH)] has been associated to this condition [Table 1]. In addition, dutasteride, a new dual 5α-R inhibitor for the treatment of BPH and androgenetic alopecia, has also been associated with this condition [Table 2].

**Table 1:** Selected reports on gynecomastia prevalence after treatment with finasteride (5 mg/day)

Number affected (%)	Comments	References
214 men reported in FDA adverse drug event surveillance	*Side affected*: 30% unilateral, 25% bilateral, 45% unspecified. Remission: 80% partial or complete remission, 20% no change. Cancer risk: One patient suspected to develop intraductal breast carcinoma due to treatment.	9
42/14722 (0.3)	Third most common side effect of finasteride. Rate of gynaecomastia was 0.26/1000 patient-months of therapy.	8
1/297 (0.3)		11
426/9423 (4.5)		12
0/70 (0)		2

**Table 2:** Selected reports on gynecomastia prevalence after treatment with dutasteride (0.5 mg/day)

Number affected (%)	References
9/813 (1)	13
50/2167 (2.3)	14
21/1188 (1.8)	15
1–1.9% annually of a 569 patient cohort	16
7/366 (2)	17
0/68 (0)	2

Gynecomastia, however, was not reported originally as a side effect in the large trial of finasteride 1 mg/day as a treatment for androgenetic alopecia,[4] and it was averred that there is no evidence that this dose causes breast tenderness or enlargement.[7] Nevertheless, several reports have described gynecomastia as a side effect of finasteride even in the lower doses [Table 3]. The time of onset (2-4 months) observed in our patients is generally in agreement with the reported literature, which shows a delay in onset of gynecomastia relative to other sexual-related adverse events.[8] Interestingly, however, when reviewing the reported cases, there is a striking prevalence of unilateral gynecomastia in the lower doses (in contrast to a similar distribution of cases between unilateral and bilateral gynecomastia with the higher doses).[9] Actually, patient 1 is the first reported case of bilateral gynecomastia after treatment with low dose finasteride. Also of importance is the fact that the breast enlargement persisted in patient 1, 10 months following finasteride withdrawal, and in the second patient residual enlargement was observed even six years following drug cessation.

**Table 3:** Reported cases of gynecomastia following finasteride (1 mg/day) *

Side	Onset time	Remission	Cancer risk	Age	References
Unilateral	4 months	Still mildly enlarged after 7 months		20	18
Unilateral	10 weeks		Cytologic atypia by FNA, but no malignancy	53	5
Unilateral	2 months	10 months	Normal mammography	18	10
Unilateral	6 months	2 months		23	10
Unilateral	3 months	3 months	Normal cytology	29	10
Unilateral	11 months	Unspecified		25	10

* Gormley *et al*. (2002) reported two patients out of 298 suffering from gynecomastia following administration of 1 mg/day of finasteride; but no further details were provided

Although a benign condition, gynecomastia may cause substantial embarrassment as well as anxiety and discomfort in the affected patient.[6] Breast tenderness and enlargement are mentioned in the drug′s leaflet, but gain no attention among practicing dermatologists who usually don′t discuss this side effect with their patients. Therefore, we believe that this side effect should be emphasized when administering this drug for the treatment of androgenetic alopecia. In addition, we share Ferrando *et al*.′s[10] opinion that this side effect is often overlooked, and that new studies are warranted in order to assess the real incidence of this side effect.
